# Nuclear dynamics in the homothallic ascomycete *Sordaria fimicola*

**DOI:** 10.1186/s40529-018-0233-y

**Published:** 2018-07-03

**Authors:** Kulsumpun Krobanan, Wei-Chiang Shen

**Affiliations:** Department of Plant Pathology and Microbiology, National Taiwan University, No. 1 Roosevelt Road, Sec. 4, Taipei, 10617 Taiwan

**Keywords:** *Sordaria fimicola*, Histone H2B protein, mCherry

## Abstract

**Background:**

The coprophilous ascomycete *Sordaria fimicola* usually reproduces sexually. Sexual differentiation in *S. fimicola* is accompanied by cellular and morphological changes, followed by multicellular tissue development to complete the sexual cycle. Although the morphological features of the sexual reproductive structure in *S. fimicola* have been well characterized, little is known about the nuclear dynamics and organization during these processes. Therefore, in this study, we successfully developed an *Agrobacterium*-mediated protoplast transformation protocol and generated histone H2B-mCherry-labeled *S. fimicola* strains. The life cycle of *S. fimicola* begins with germination of the ascospore and ends with the formation and discharge of new ascospores from the mature black sexual fruiting bodies, the so-called perithecia. The nuclear dynamics of the fluorescently labeled strains were examined during ascospore germination, hyphal elongation, and hyphal fusion using fluorescent microscopy.

**Results:**

Live imaging revealed that the nuclei in the germlings and fusion hyphae during the pre-contact interaction are located adjacent to the tip.

**Conclusions:**

This is the first report of the application of a fluorescence labeling technique in *S. fimicola.* This application will help researchers gain a better understanding of nuclear distribution and investigate the protein–protein interaction networks during fruiting body formation for advanced molecular genetic studies in *S. fimicola.*

**Electronic supplementary material:**

The online version of this article (10.1186/s40529-018-0233-y) contains supplementary material, which is available to authorized users.

## Background

*Sordaria fimicola* is a well-known homothallic ascomycete commonly found in fecal matter and has long been used to study genetic recombination, chromosome segregation, and the fungal life cycle as a whole (Ingold and Dring 1957; Olive 1956). *Sordaria fimicola* is easily cultivated in artificial cultures and can undergo sexual reproduction with a short life cycle under laboratory conditions (Olive 1956). The *S. fimicola* life cycle starts with mature sexual spores released from fruiting bodies embedded in animal dung, and spore germination is known to be induced by certain stimulants including sodium acetate (Bretzloff 1951). Once germinated, self-fertilization between hyphae in the same colony preferably initiates the sexual development. During hyphal fusion, pre-contact and contact interactions are observed between compatible hyphae, and the dissolution of cell walls allows nuclei to migrate to partner hyphae. The stages of coli formation followed by ascogenous hyphae differentiation have been reported (Piehl 1929). The multinucleate ascogenous hyphae then undergo septation to form binucleate penultimate cells where nuclear fusion occurs, followed by the subsequent development into asci. The young asci initially show lateral outgrowth and further elongation but maintain the primary nucleus before division occurs (Piehl 1929). Then, meiosis, one round of mitosis, and spore wall formation eventually lead to the production of eight haploid ascospores in a linear arrangement (Olive 1956; Piehl 1929). Coordination of these developmental events with tissue-specific differentiation leads to the final production of fruiting bodies, called perithecia, with mature asci and ascospores enclosed. Although the morphological and cytological features of the sexual structures in *S. fimicola* have been well described, the nuclear organization and dynamics during these processes remain unclear.

Fluorescently nuclear-tagged strains have been used to investigate the nuclear behavior and dynamics of live cells in many fungal systems such as the nuclear dynamics and their associated microtubules in *Neurospora crassa* (Freitag et al. 2004; Roca et al. 2010) and the nuclear dynamics during spore germination and hyphal fusion in *Fusarium oxysporum* (Ruiz-Roldan et al. 2010). Recently, the polar outgrowth and branching of young hyphae that emerge from the germination vesicles of ascospores were reported in *Sordaria macrospora* (Teichert 2017). Although *S. fimicola* has long been used for various aspects of fungal research (Ingold and Dring 1957; Ingold and Hadland 1959; Kitani and Whitehou 1974; Olive 1956), no strain generated via a genetic transformation method has yet been established for this organism to date. To expand the capabilities of *S. fimicola* in genetic analyses, in this study, we conducted the first successful transformation of *S. fimicola* and generated a histone H2B-mCherry-labeled strain, which was used to examine the nuclear dynamics during ascospore germination, sexual differentiation, and hyphal extension. In addition to providing a useful tool for genetic research, our time-lapse video of the nuclear dynamics should facilitate a better understanding of the detailed nuclear behaviors that occur during the life cycle and reproductive events in *S. fimicola*.

## Methods

### Strains, media, and culture conditions

*Sordaria fimicola* (Roberge ex Desm.) Ces. & De Not. (BCRC 33665) was obtained from the Bioresource Collection and Research Center (BCRC), Hsinchu, Taiwan and used as the experimental research strain in this study. Cultures of *S. fimicola* were grown on malt extract agar I [Blakeslee’s Formula; 2% malt extract (Himedia, India), 2% glucose, 0.1% peptone and 1.5% (or 2%) agar], and malt extract agar III [2% malt extract and 1.5% (or 2%) agar] was used to induce the formation of sexual reproductive structures. The compositions of malt extract agar I and III media were provided by the BCRC website as previously reported (Lin et al. 2006). *Escherichia coli* DH5α and *Agrobacterium tumefaciens* EHA105 strains were routinely maintained on Luria–Bertani (LB) medium (Sambrook and Russell 2001). *E. coli* and *A. tumefaciens* transformants harboring targeted plasmids were selected on LB containing either 100 µg/ml ampicillin or 100 µg/ml kanamycin.

### Nucleic acid manipulation and analysis

Genomic DNA (gDNA) of *S. fimicola* was extracted following slight modifications of a previously described protocol (Pitkin et al. 1996). The strains were grown on cellophane-covered malt extract agar I medium, and mycelia grown on the cellophane were peeled and dipped into liquid nitrogen. The mycelial pad was ground into powder using a mortar and pestle with liquid nitrogen. The fine powder was homogenized in 500 µl DNA extraction buffer [l00 mM Tris/HCl, pH 7.5, 2% hexadecyltrimethylammonium bromide (CTAB), 1.4 M NaCl, 20 mM ethylenediaminetetraacetic acid (EDTA), 2% polyvinylpyrrolidone (PVP-40) and 1% mercaptoethanol] and then incubated for 1 h at 65 °C. The cell lysate was extracted with the same volume of phenol:chloroform (1:1) and centrifuged at 13,200 rpm, 4 °C for 15 min, and the supernatants were saved for subsequent chloroform extraction. DNA was precipitated by adding isopropanol and incubated for 15 min at 25 °C. DNA was harvested by centrifuging at 12,000 rpm, 4 °C for 10 min. The pellet was washed with 70% ethanol and collected by centrifugation. The DNA pellet was air-dried and re-suspended in sterile distilled water.

### *Sordaria fimicola* transformation

*Sordaria fimicola* transformation was carried out using an *Agrobacterium*-mediated protoplast approach with slight modification (Idnurm et al. 2004; Minz and Sharon 2010; Walz and Kuck 1993). In brief, the mycelial plugs were grown in 100 ml malt extract broth I or complete liquid medium containing 1% glucose, 0.2% tryptone, 0.2% yeast extract, 0.15% KH_2_PO_4_, 0.05% KCl, 0.05% MgSO_4_, 0.37% NH_4_Cl, and 1 ml/l of a solution containing 1% of ZnSO_4_, Fe(II)Cl_2_, and MnCl_2_ at 28 °C in 500-ml Erlenmeyer flasks and then further incubated at 28 °C for 3 days without shaking (Nowrousian et al. 1999). The mycelia homogenized in a blender were recovered for 1–2 h at 28 °C with shaking at 80–100 rpm. Recovered mycelia were harvested by centrifugation and subsequently washed with protoplast buffer (0.01 M Tris, 0.01 M MgSO_4_·7H_2_O, 1 M KCl, pH 7.0). Protoplasts were generated by digesting with lysing buffer composed of 20 mg/ml of VinoTaste^®^ Pro enzyme (Novozymes, Denmark), 2 mg/ml β-d-glucanase (InterSpex Product, USA), 5 mg/ml Driselase (InterSpex Product, USA), and 0.47 µl/ml of β-glucuronidase (Sigma, USA) in protoplast buffer at 28 °C for 12 h. To obtain protoplast cells, the lysate solution was filtered through Miracloth (Calbiochem, Germany), washed with protoplast buffer, collected by low-speed (1000–1200 rpm) centrifugation for 15 min at 4 °C, and adjusted to a final concentration of 10^7^–10^8^ cells/ml (Walz and Kuck 1993).

The *A. tumefaciens* EHA105 strain was used as the host strain to deliver the target gene to *S. fimicola* according to a slightly modified *Agrobacterium* mediated protoplast transformation protocol (Idnurm et al. 2004; Minz and Sharon 2010). First, *Agrobacterium* was subjected to plasmid electroporation. The *Agrobacterium* strain was pre-cultured in LB liquid medium supplemented with 100 µg/ml of kanamycin at 28 °C for 48 h. Bacterial cells were washed in minimal medium and then re-suspended in induction medium (minimal medium supplemented with 100 µM acetosyringone) until reaching an optical density of 0.15 at 660 nm. The culture was further incubated with shaking for 6 h at 28 °C, and then 200 µl of *A. tumefaciens* cells (in protoplast buffer) was added to 200 µl of the *S. fimicola* protoplasts obtained as described above (10^7^–10^8^ cells/ml). The mixtures were plated on induction medium agar (containing 5 mM glucose and 200 µM acetosyringone). After incubation at 28 °C for 48 h, the induction medium agar plates were washed with 5 ml of malt extract I or CM liquid medium, and 1 ml was plated onto corn meal agar medium supplemented with 200 µg/ml cefotaxime and 100 µg/ml hygromycin and incubated at 28 °C for 2 days. The colonies of the transformants were transferred to malt extract agar I meal agar medium containing 100 µg/ml hygromycin B.

### Generation of fluorescence plasmids

To construct a nuclear fluorescence plasmid, the PR27p-H2B-mCherry plasmid was amplified by fusion polymerase chain reaction (PCR) using KAPA HiFi DNA Polymerase (KAPA Biosystems, USA) according to the manufacturer’s protocol. The *PR27* promoter and *TrpC* terminator fragments were amplified with the primer pairs WC1218/WC2902 and WC2905/WC2906, respectively. *S. fimicola* histone H2B cDNA and mCherry fragments were then obtained by amplification with WC2903/WC2904 and WC601/WC602, respectively. The amplified fragments were cleaned with QIAquick Gel Extraction Kit and QIAquick PCR Purification Kit (QIAGEN, Germany). The fusion fragment of PR27p-H2B and mCherry-TrpCt was obtained by amplification with WC1218/WC2904 and WC601/WC2906, respectively. These two fragments were purified, mixed, and amplified with primers WC1218 and WC2906 to generate the PR27p-H2B-mCherry-TrpCt fragment. The overlap-extension PCR product was cloned into the pCR^®^-BluntII-TOPO^®^ vector (Invitrogen, USA) and subsequently subcloned into pCAMBIA::hyg(S) by cutting with SpeI and SmaI to finally obtain pCAMBIA::hyg::RP27p-H2B-mCherry-TrpCt. The pCAMBIA::hyg(S) plasmid was generated by cutting with XhoI and SacI and ligating with a 1.4-kb *hph* cassette from pCR::hyg derived from the pCB1004 vector. For PCR screening, the reaction was carried out using BioTaq DNA Polymerase (Bioman Scientific, Taiwan). Primer sequences and strains are listed in Tables 1 and 2, respectively.

**Table 1:** Oligonucleotide primers used in this study

Primer	Sequence (5′–3′)	Purpose
WC33	TCCGTAGGTGAACCTGCGG	ITS1F
WC35	TCCTCCGCTTATTGATATGC	ITS4R
WC601	ATGGTGAGCAAGGGCGAGGAGGATAA	mCherryF
WC602	CTACTTGTACAGCTCGTCCATGCC	mCherryR
WC1218	CAGGTCTACAACCGTGATTA	PR27pF
WC2902	TTTGAAGATTGGGTTCCTAC	PR27pR
WC2903	GTAGGAACCCAATCTTCAAAATGCCTCCCAAGCCCGCCGAC	SfcH2BF
WC2904	TCCTCCTCGCCCTTGCTCACCATTTTCGTGCTGGAGGAGTACTTG	SfcH2BR
WC2905	CATGGACGAGCTGTACAAGTAG CACTTAACGTTACTGAAATCAT	TrpCF
WC2906	GATCCTCTAGAAAGAAGGAT	TrpCR

**Table 2:** Strains used in this study

Strain	Genotypes	Source
*A. tumefaciens* EHA105	Wild type	Laboratory
*S. fimicola* BCRC 33665	Wild type	BCRC
*S. fimicola* WTH2B/2	WT:: RP27p-H2B-mCherry	This study
*S. fimicola* WTH2B/3	WT:: RP27p-H2B-mCherry	This study
*S. fimicola* WTH2B/4^a^	WT:: RP27p-H2B-mCherry	This study
*S. fimicola* WTH2B/5	WT:: RP27p-H2B-mCherry	This study
*S. fimicola* WTH2B/6	WT:: RP27p-H2B-mCherry	This study

*BCRC* Bioresource Collection and Research Center

^a^Used for fluorescent observation

### Microscopic observations

The ascospores of the *S. fimicola* wild-type and fluorescently labeled strains were inoculated in the center of a malt extract agar III plate (6-cm Petri dish) and incubated at 28 °C under uniform constant white light. The cultures were observed based on the time course of the developmental structure, as shown in Table 3. For fluorescence microscopy, the images and time-lapse movies of live cells were captured with the Deltavision core system (Applied Precision, USA) equipped with an EMCCD (Electron-multiplying CCD) camera (Photometrics Evolve^™^ 512) on an Olympus IX71 inverted microscope, using UPlanSApo 100×/1.40 oil, UApo, 40×/1.35 oil, and UPlanSApo 20×/0.75 objective lenses. The mCherry fluorescence images were acquired using standard filter sets of 575/25 and 632/60 nm for excitation and emission, respectively. Images were analyzed using SoftWoRx-Acquire version 5.5.1 (Applied Precision, USA).

**Table 3:** Time courses used in this study

Day post inoculation (days)	Hour after inoculation (h)	Developmental stage
	2, 3 and 6	Germination
	15	Hyphal elongation and fusion
1	27	Ascogonial coils formation
2	48	Protoperithecia formation
4	192	Mature perithecia with black ascospores

## Results and discussion

### Generation of the nuclear-labeled strains

**Fig. 1:**
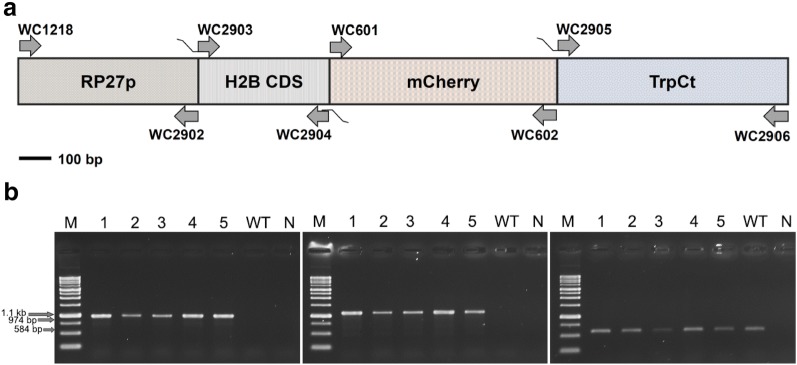
Generation of fluorescently labeled *S. fimicola* strains. **a** Schematic presentation of the plasmid construct used to generate histone H2B-mCherry-labeled strains. **b** PCR was used to verify the successful generation of the H2B-mCherry-labeled strains. The plasmid construct was ectopically integrated into the *S. fimicola* wild type strain (WT: BCRC33665). The transformants and wild-type strain are shown in Lanes 1–5 and WT, respectively. Amplification with primer pairs WC1218/WC2904 (left), WC2903/WC602 (middle), and ITS1/ITS4 (right) generated fragments of 974 bp, 1.1 kb, and 584 bp, respectively

To examine nuclear dynamics in *S. fimicola*, we aimed to generate fluorescent, nuclear-labeled strains to record live images of the nuclear distribution and behavior during different stages of the life cycle. Since histones are essential structural proteins for chromatin assembly and play functional roles in all living cell types, we used the fluorescent mCherry protein to tag H2B histone to monitor nuclear distribution in the ascospore germlings, vegetative hyphae, and initial fusing and other sexual structures. As shown in Fig. 1, we successfully generated the histone H2B-labeled strains using *Agrobacterium*-mediated protoplast transformation. Observation of the hygromycin B-resistant transformants under the fluorescent microscope revealed that five transformants clearly displayed strong mCherry signals. These transformants were further verified by PCR to detect the presence of the PR27p-H2B-mCherry fragment. Indeed, 974-bp and 1.1-kb fragments were amplified from these transformants with the primer pairs WC1218/WC2904 and WC2903/WC602, respectively, whereas no PCR product was obtained from the wild type (Fig. 1b, left and middle). Amplification of gDNA with the universal ITS1/ITS4 primer pair as a control resulted in a 584-bp product in both the wild-type and transformed strains (Fig. 1b right). Based on these results, the transformant WTH2B/4 was chosen for further investigation of nuclear dynamics.

### Nuclear distribution during ascospore germination in *S. fimicola*

**Fig. 2:**
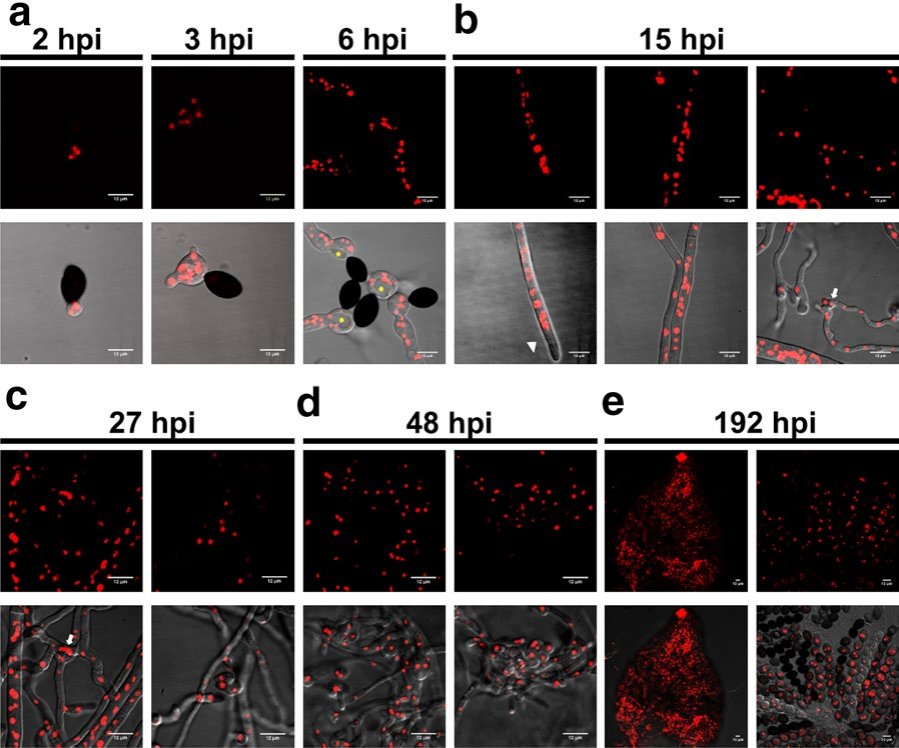
Live-cell imaging of nuclear distribution during the *S. fimicola* life cycle. The H2B-mCherry-labeled strain was cultured on malt extract agar III under constant white light. The nuclear distribution in a single germination vesicle and germ tube from an ascospore was observed at 2, 3, and 6 h post-inoculation (hpi). At 15 hpi, nuclear fluorescence was distributed in the mature vegetative hyphae and hyphal fusion regions, whereas the nuclei were not detected in the hyphal tip regions of the mature vegetative hyphae. Nuclear distribution in the coil and ascogonia hyphae, including the early protoperithecia, was detected within 27 and 48 hpi, respectively. At approximately 192 hpi, mature perithecia with asci containing black mature and multinucleated ascospores were observed. Live images were captured with the Deltavision core system. Nuclear distribution in each developmental stages of *S. fimicola* including germination vesical formation (**a**), hyphal extension and contact (**b**), ascogonial coils formation (**c**), differentiated ascogenous hyphae development (protoperithecia) (**d**) and perithecia formation (**e**) are showed. White arrows and arrowheads indicate hyphal fusion and the extension tip of a vegetative hypha, respectively. Yellow asterisks indicate vacuoles. Scale bars represent 12 μm

**Fig. 3:**
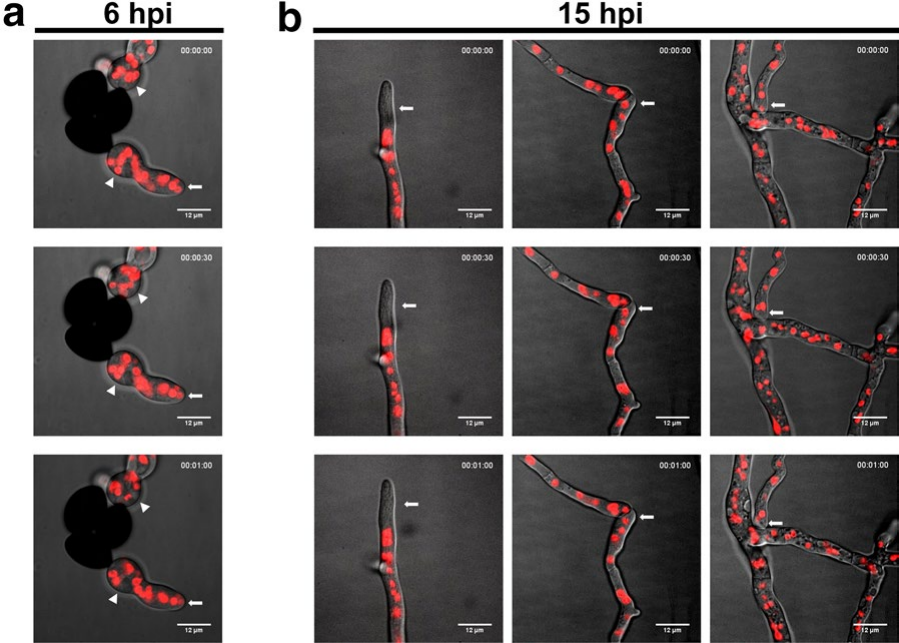
Nuclear distribution of germ tube and mature vegetative hyphal tip and representative hyphal fusion events. The ascospores of the H2B-mCherry-labeled strain were grown on malt extract agar III under uniform constant white light. **a** Nuclear florescence was evenly distributed throughout the germ tube at 6 h post-inoculation (hpi). **b** At 15 hpi, the nuclei in the elongated vegetative hyphae (left) were absent in the tip regions, whereas nuclei were detected in the apical regions of pre-fusion hyphae [either tip-to-tip (middle) or tip-to-side (right)]. Live images were captured with the Deltavision core system. White arrows indicate differentiated sites of nuclear distribution among these fungal structures. White arrowheads indicate germination vesicles. Scale bars represent 12 μm

To examine the nuclear dynamics in different sexual structures, the ascospores of *S. fimicola* strains were first collected and inoculated onto malt extract agar III. Approximately 1–2 h post-inoculation, the ascospores of the H2B-mCherry-labeled strain formed a germination pore at one end of the spore, followed by the formation of the germination vesicle, which allowed the nuclei to enter the vesicle (Fig. 2). This differs from the germination behavior of *N. crassa*, whose ascospore was reported to germinate from one or both ends through the germ pore (Seale 1973), but is similar to the germination of *N. terricola,* which is mostly restricted to a single germ pore (Cai et al. 2006). The germ pore or emergence of the germination vesicle appeared to be located at the pointed terminus of the ascospore, while the opposite side of the germination pore seemed more rounded, indicating that *S. fimicola* has only one polar germ pore. Moreover, the ascospores in *Sordaria* spp. have been previously found to share common morphological characteristics, including smooth walls with a basal single germ pore and gelatinous sheath (Cai et al. 2006). Physically, the germination of *S. fimicola* ascospores does not require any activation stimuli, which is similar to the situation for *S. macrospora* ascospores (Teichert 2017). In contrast, *N. crassa* germination requires either heat shock, a chemical agent, or both to activate the dormant ascospores (Emerson 1948, 1954). Moreover, the expansion of germination vacuoles was observed in the cytoplasm of the germination vesicle during the outgrowth of the germ tube, and the spore was eventually found to be empty (Hackett and Chen 1976). At 6 h post-inoculation, various inclusions were visible in this germling, including a single large or multiple medium-sized lipid droplet vacuoles in the center of the germination vesicle. These structures likely reflect vacuole inclusions that might help push nuclei toward the germination vesicle and the peripheral growth zone of a fungal colony and/or serve as nutrient reservoirs during establishment on solid agar (Figs. 2, 3; Additional file 1: Video S1). Interestingly, lipid droplet vacuoles in the germinating ascospores of *S. brevicollis* and *N. tetrasperma* have been similarly reported (Hackett and Chen 1976; Lowry 1968).

### Nuclear behavior in the vegetative hyphae

After germination, the germ tube emerging from the vesicle continued elongating and branching to produce young vegetative hyphae. Initially, a uniform nuclear distribution was displayed along the length of the vegetative hyphae (Figs. 2, 3; Additional file 1: Video S1). However, at 15 h post-inoculation, the hyphal tips were devoid of nuclei (Figs. 2, 3; Additional file 2: Video S2). Such a nuclei-devoid region near the hyphal tip has also been described in other fungal systems, including *N. crassa* (Araujo-Palomares et al. 2007) and *S. macrospora* (Teichert 2017). This phenomenon is generally explained by the presence of a mature Spitzenkörper structure formed at the hyphal tip region, indicating the transition from a germling to vegetative hypha (Araujo-Palomares et al. 2007).

The pre-fusion interaction between vegetative hyphae was also observed at 15 h post-inoculation (Figs. 2, 3). The main morphology types of the hyphal fusions were tip-to-side and tip-to-tip. The hyphal tips approached other fusion-competent hyphae by growing toward their partners via a Spitzenkörper mechanism, whereby the approaching hyphal tips also made contact with other partner hyphae, and subsequently apical extension ceased to prevent protoplasmic flow through the fusion pore (Hickey et al. 2002). This observation indicated that the hyphal tip extension phase was replaced by hyphal orientation to form the mating structure (Brand and Gow 2009). Some of the approaching hyphae showed slight swelling of the apex but also displayed nuclei at the tip ends (Fig. 3), indicating that the end of Spitzenkörper function in the apical region resulted in a physiological change of the hyphal tip and allowed for the presence of nuclei.

### Nuclear distribution during sexual development in *S. fimicola*

As sexual development further proceeded, ascogonial coils, likely formed as side branches of vegetative hyphae ca. 8–10 μm wide, were observed at 27 and 48 h post-inoculation (Fig. 2). At 48 h post-inoculation, in addition to ascogonial coil formation, multinucleate cellular compartments resulting from differentiated ascogenous hyphae were observed in this culture and defined as early protoperithecia (Fig. 2). These structures were ca. 30 μm in width and showed the packing of various hyphae. In fact, the protoperithecia developed from the ascogonium were formed by the aggregation of enveloping hyphae that emerged from either the ascogonial coil or neighboring hyphae, as previously described in detail (Lord and Read 2011). Through the growth of the protoperithecium accompanied by septation and tissue differentiation, the dark-pigmented mature fruiting body (perithecium) with its neck was observed at approximately 192 h post-inoculation, with a size of ca. 250–300 μm wide. Moreover, an ascus rosette containing asci of various stages of maturity derived from the perithecium of the mCherry-labeled strain was observed. The black, mature ascospore contained multiple nuclei derived from repetitive mitotic division, as shown in Fig. 2 (192 h post-inoculation).

## Conclusions

Overall, in the present study, we established a gene delivery system for *S. fimicola* that allowed us to examine the nuclear dynamics that occurs during spore germination and the different stages of sexual development. Although much is known about nuclear distribution during hyphal extension and fusion in fungi such as *N. crassa* and *F. oxysporum,* little is known about hyphal fusion in self-fertilized fungi such as *S. fimicola*. Research on nuclear behavior using this tool could help unveil the complex pre- and post-fusion mechanisms, which would in turn provide valuable insights for understanding cell fusion in other eukaryotic species. Moreover, the link between the hyphal fusion event and fruiting body development is one of the most important questions in fungal biology, and the model genetic system of *S. fimicola* can be applied to further understand the fundamental processes in the sexual reproduction of eukaryotic cells.

## Additional files

**Additional file 1: Video S1.** Nuclear behavior in the hyphal cells of H2B-mCherry-labeled strain. Nuclear trafficking in a germ tube emerging from a single germ pore displayed a uniform distribution throughout the structure. Scale bar represents 12 µm. The video shows a period of 20 min with a time lapse of two frames per minute.

**Additional file 2: Video S2.** Nuclei absent from the extension tip regions of vegetative hyphae. Scale bar represents 12 µm. The video shows a period of 20 min with a time lapse of two frames per minute.

## Abbreviations

BCRC: Bioresource Collection and Research Center
LB: Luria–Bertani
CTAB: hexadecyltrimethylammonium bromide
EDTA: ethylenediaminetetraacetic acid
PVP-40: polyvinylpyrrolidone-40
EMCCD: electron-multiplying CCD
PCR: polymerase chain reaction
CM: complete medium
ITS1/ITS4: internal transcribed spacer 1/4 primers of nuclear ribosomal DNA
